# TRAIL inhibits RANK signaling and suppresses osteoclast activation via inhibiting lipid raft assembly and TRAF6 recruitment

**DOI:** 10.1038/s41419-019-1353-3

**Published:** 2019-01-28

**Authors:** Hsiu-Jung Liao, Hwei-Fang Tsai, Chien-Sheng Wu, I.-Tsu Chyuan, Ping-Ning Hsu

**Affiliations:** 1 Graduate Institute of Immunology, College of Medicine, National Taiwan University, Taipei, Taiwan; 2 Department of Internal Medicine, Taipei Medical University Shuang Ho Hospital, New Taipei City, Taiwan; 3 Graduate Institute of Clinical Medicine, College of Medicine, Taipei Medical University, Taipei, Taiwan; 4 Division of Rheumatology, Department of Internal Medicine, Far Eastern Memorial Hospital, New Taipei City, Taiwan; 5 Department of Internal Medicine, Cathay General Hospital, Taipei, Taiwan; 6 School of Medicine, College of Medicine, Fu Jen Catholic University, New Taipei City, Taiwan; 7 Department of Internal Medicine, National Taiwan University Hospital, Taipei, Taiwan

## Abstract

Human osteoclast formation from mononuclear phagocyte precursors involves interactions between members of the tumor necrosis factor (TNF) ligand superfamily and their receptors. Recent evidence indicated that TNF-α-related apoptosis-inducing ligand (TRAIL) induces osteoclast differentiation via a TRAF6-dependent signaling pathway; but paradoxically, it inhibits RANK ligand (RANKL)-induced osteoclast differentiation. Although a number of signaling pathways were linked to the RANK and osteoclastogenesis, it is not known how TRAIL regulates RANK signaling. In this study, we demonstrate that TRAIL regulates RANK-induced osteoclastogenesis in terms of the assembly of lipid raft-associated signaling complexes. RANKL stimulation induced recruitment of TRAF6, c-Src, and DAP-12 into lipid rafts. However, the RANKL-induced assembly of lipid raft-associated signaling complexes and TRAF6 recruitment was abolished in the presence of TRAIL. TRAIL-induced dissociation of RANKL-induced lipid raft signaling complexes was reversed by treatment with TRAIL receptor (TRAIL-R) siRNA or an anti-TRAIL-R blocking antibody, indicating that TRAIL mediates suppression of RANKL-induced lipid raft signaling via interactions with TRAIL-R. Finally, we demonstrated that TRAIL suppressed inflammation-induced bone resorption and osteoclastogenesis in a collagen-induced arthritis (CIA) rat animal model. Our results provide a novel apoptosis-independent role of TRAIL in regulating RANK signaling and suppresses osteoclast activation via inhibiting lipid raft assembly and TRAF6 recruitment.

## Introduction

Osteoclasts are multinucleated cells, derived from precursors of monocyte/macrophage lineages, and are specialized for bone absorption and remodeling. It is already known that normal differentiation of osteoclasts requires TNF family receptors, such as RANK^1–5^. RANK provokes biochemical signaling via the recruitment of intracellular TNF receptor-associated factors (TRAFs) after ligand binding and receptor oligomerization. Accumulating evidence from various laboratories indicates that TRAFs, most importantly TRAF6, are key to understanding how RANK ligand (RANKL) links cytoplasmic signaling to the nuclear transcriptional program^6–9^. It is likely that the RANK/RANKL/osteoprotegerin (OPG) system is the central and primary regulator of bone remodeling; however, it is clear that this is not the only mechanism involved. Many of the cytokines and growth factors implicated in inflammatory processes in inflammatory diseases were also demonstrated to impact osteoclast differentiation and function either directly, by acting on cells of the osteoclast lineage, or indirectly, by acting on other cell types that modulate expressions of the key osteoclastogenic factor, RANKL, and/or its inhibitor, OPG^10–13^. In addition to RANKL, recent studies demonstrated there are several TNF family molecules which promote osteoclast differentiation, including TNF^14^, FasL^15^, decoy receptor 3 (DcR3)^16^, and TRAIL^17,18^, indicating that activated T cells and inflammatory responses can remodel bone homeostasis via these effector molecules. TRAIL, a member of the TNF ligand superfamily, induces apoptosis in diverse tumor cell lines^19^, and its expression is upregulated in activated T cells. Previous studies demonstrated that in addition to triggering apoptosis, TRAIL induces osteoclast differentiation in mononuclear phagocyte precursors^17,18^, and is also able to suppress osteoclastic differentiation induced by RANKL plus M-CSF^20^, suggesting that TRAIL may play a role in regulating osteoclast differentiation, and this may implicate it in osteoimmunology in immune response-associated bone absorption. However, the mechanism and signaling pathways of how TRAIL regulates RANKL-induced osteoclast differentiation are still not clear.

Rafts are specialized membrane microdomains enriched in cholesterol, glycosphingolipids, and glycosylphosphatidylinositol (GPI)-anchored proteins^21,22^. Lipid rafts are dynamic assemblies of proteins and lipids that harbor many receptors and regulatory molecules, and so act as a platform for signal transduction. In T cell antigen receptor signaling, raft domains function as signaling platforms where selective signaling molecules are recruited^23,24^, which initiate downstream signaling cascades by phosphorylating tyrosine residues on receptor complexes. It was demonstrated that RANK-mediated signaling and osteoclast function are dependent on the expression and integrity of lipid rafts^25^. It is still not clear whether lipid raft-associated signaling is critical for RANK signaling, or whether TRAIL regulates RANK signaling at lipid raft-associated signaling. To understand the TRAIL-mediated regulation of RANK signal transduction osteoclastogenesis, we studied the roles of lipid raft-associated signaling in RANKL-induced osteoclast differentiation and bone resorption. We demonstrated that RANKL stimulation induced recruitment of TRAF6, c-Src, and DAP-12 into lipid rafts. However, the RANKL-induced assembly of lipid raft-associated signaling complexes was abolished in the presence of TRAIL. Our results indicated that lipid raft-associated signaling is essential for RANKL-induced osteoclast differentiation, and TRAIL inhibits RANK signaling and suppresses osteoclast activation via inhibiting lipid raft assembly and TRAF6 recruitment. Our study results suggest that TRAIL modifies RANK signaling by interacting with lipid raft-associated signaling. This provides new insights into the molecular mechanism that may implicate osteoimmunology in the immune response associated with bone absorption.

## Materials and Methods

### Animals

Sprague–Dawley (SD) rats (male, 6–8 weeks old) and C57BL/6 mice (male, 6–8 weeks old) were housed under specific pathogen-free conditions and provided with standard food and water. TRAIL receptor (TRAIL-R) knockout (*Trail-r*^−/−^) mice were obtained from Henning Walczak (UCL Cancer Institute, University College London, UK). All animal work was conducted according to guidelines of the Association for Assessment and Accreditation of Laboratory Animal Care. All animal experiments were approved by the Animal Ethics Committee of the National Taiwan University Medical Center.

### Induction of collagen-induced arthritis (CIA) and assessment of arthritis

Bovine collagen type II (bCII) was solubilized at 2 mg/ml in 0.05 M acetic acid, and then emulsified in an equal volume of complete Freund’s adjuvant (CFA) containing 4 mg/ml of heat-killed *Mycobacterium tuberculosis* (Arthrogen-CIA; Chondrex, Redmond, WA) in an ice-cold water bath. Male SD rats were first subcutaneously immunized (day 0) at the base of the tail with 0.2 ml of this emulsion. On day 7, rats were given a booster injection of 0.2 ml of the emulsion. Clinical signs of arthritis in each hind paw were assessed under blinded conditions every day. The hind paw thickness was measured with Vernier calipers. The severity of arthritis in each hind paw was monitored and scored according to the following criteria: 0 = no evidence of erythema and swelling; 1 = erythema and mild swelling confined to the tarsals or ankle joint; 2 = erythema and mild swelling extending from the ankle to the tarsals; 3 = erythema and moderate swelling extending from the ankle to metatarsal joints; and 4 = erythema and severe swelling encompassing the ankle, foot, and digits. Each hind paw was graded and an average score of ≥ 3 was defined as severe arthritis.

### Reagents

Recombinant murine TRAIL was purchased from PeproTech (Rocky Hill, NJ) and diluted with phosphate-buffered saline (PBS) to produce a dose of 1.5 μg/kg body weight (BW)/rat. The soluble TNF-α receptor, etanercept (Enbrel), was purchased from Pfizer (New York City, NY) and diluted with PBS to produce a dose of 6 mg/kg BW/rat. Mouse immunoglobulin G (IgG) was purchased from R&D Systems (Minneapolis, MN) and diluted with PBS to produce a dose of 200 ng/rat. Rats were injected subcutaneously at the hind paws with PBS, IgG, TRAIL, or etanercept starting on day 4 after the booster immunization (day 11 after the bCII immunization).

### Cell cultures and treatments

We used bone marrow-derived macrophages (BMMs) and the RAW 264.7 murine monocytic/macrophagic cell line as model systems of osteoclastogenesis. Both cell types differentiate into osteoclast-like cells in the presence of RANKL plus M-CSF. Bone marrow cells from C57BL/6 mice were cultured in minimum essential medium (MEM) supplemented with 50 ng/ml M-CSF, 10% FCS, 2 nM L-glutamine, and 100 U/ml penicillin/streptomycin for 3 days. Adherent cells were referred to as BMMs. For induction of osteoclast differentiation, RAW 264.7 cells, mouse macrophage cell line, and BMMs were seeded in a 96-well plate at a density of 2 × 10^5^/cells and cultured for 7 days in the presence of 100 ng/ml RANKL and 50 ng/ml M-CSF, or 100 ng/ml TRAIL. The medium was replaced every three days.

### TRAIL receptor (TRAIL-R) small interfering (si)RNA transfection

siRNA oligonucleotides against TRAIL messenger (m)RNA were purchased from Dharmacon (Thermo Scientific, Dharmacon, CO). siRNA oligos were resuspended and diluted to a concentration of 80 nmol/ml. RAW 264.7 cells or BMMs from mice were transfected with 4 nmol siRNA oligo using a Bio-Rad Gene Pulser II system at 280 V and 975 μF.

### Tartrate-resistant acid phosphatase (TRAP) staining

Cells were fixed and stained with TRAP (Sigma-Aldrich, St. Louis, MO) for 1 h at 37 °C, followed by counterstaining with a hematoxylin solution. TRAP^+^ multinuclear cells (MNCs) (with more than three nuclei) were regarded as osteoclasts and counted under an inverted-phase contrast microscope. Morphological features of osteoclasts were photographed with a photomicroscope. For detecting the osteoclasts in the tissue sections, osteoclast numbers were measured by quantifying cells positively stained for TRAP (Sigma-Aldrich, St. Louis, MO). Briefly, specimens were fixed for 30 s and then stained with naphthol AS-BI phosphate and a tartrate solution for 1 h at 37 °C, followed by counterstaining with a hematoxylin solution. TRAP-positive multinuclear cells with three or more nuclei were regarded as osteoclasts and counted under an inverted-phase contrast microscope. The total number of TRAP-positive cells and the number of nuclei per TRAP-positive cells in each well were counted.

### Isolation of nuclei for detecting NFATc1

To detect activation of NFATc1, RAW 264.7 cells were treated with the TRAIL (500 ng/ml), M-CSF (20 ng/ml), and RANKL (50 ng/ml) in the presence or absence of TRAIL-R siRNA or anti-TRAIL-R. After stimulation, cell lysates of the nuclear fraction were prepared. Nuclear and cytosolic proteins were separated upon resuspension of pelleted cells (10^6^) in 30 ml of hypotonic lysis buffer (10 mM Tris-HCl [pH 7.4], 10 mM NaCl, 3 mM MgCl_2_, and 0.5% NP-40) by gentle pipetting, then incubation on ice for 5 min. The homogenate was centrifuged at 6000 rpm for 5 min at 4 °C. The supernatant representing the cytosolic fraction was collected and stored at –80 °C, and the pellet containing cell nuclei was lysed in 30 ml RIPA lysis buffer. The nuclear extract (supernatant) was retained after centrifugation at 13,000 × *g* for 5 min at 4 °C for subsequent western blotting with anti-NFATc1 (Thermo Scientific, Rockford, IL) and anti-histone deacetylase (HDAC) antibodies (Cell Signaling, Danvers, MA).

### Lipid raft and non-lipid raft fractionation for protein analysis

Lipid rafts are defined by their solubility in cold, non-ionic detergent. Stimulated RAW cells (5 × 10^7^) were treated with 375 μl of cold lysis TNE buffer (TNE buffer: 25 mM Tris [pH 7.5], 150 mM NaCl, 5 mM EDTA, 1 × protease inhibitor, and 1 × phosphatase inhibitor) and incubated for 30 min on ice. TNE buffer containing 0.5% Brij-58 was used for TRAF6 and c-Src isolation. Lysates were then mixed with 375 μl of 85% sucrose in TNE buffer. The solution was overlaid with 2.25 ml of 30% sucrose in TNE buffer, followed by 1.5 ml of 5% sucrose in TNE buffer. The sucrose gradients were centrifuged at 2 × 10^5^× *g* in a SW55Ti rotor (Beckman Coulter, Fullerton, CA) overnight at 4 °C. Twelve fractions of proteins were added to sodium dodecylsulfate (SDS) sample buffer and resolved by SDS-polyacrylamide gel electrophoresis (PAGE) and Western blotting.

### Western blot analyses

To detect activation of cytosolic and lipid raft signaling molecules, RAW 264.7 cells or mouse BMMs were stimulated with the RANKL or TRAIL. Whole cell lysates were transferred and immunoblotted with specific antibodies for RANK, TRAF6, Src, DAP-12, and anti- flotillin (Cell Signaling Technology, Danvers, MA). For nuclear translocation of transcription factors, stimulated cells were lysed with hypotonic buffer and fractionated into cytoplasmic or nuclear fractions, followed by immunoblotting with specific antibodies for NFATc1 and HDAC1 (Cell Signaling Technology, Danvers, MA).

### Immunofluorescence confocal microscopy

RAW 264.7 cells (10^5^) were cultured on coverslips. Cells were cultured with M-CSF for 1 day and treated with RANKL for another 4 h. After washing, cells were incubated with PE-conjugated cholera toxin (Ctx) B subunit for 30 min at 4 °C. Cells were fixed with 4% formaldehyde for 30 min and washed with 1% bovine serum albumin (BSA)/PBS. Cells were then permeabilized with 0.5% saponin in 1% BSA dissolved in PBS for 30 min. Cells were incubated with an anti-RANK, anti-TRAF6 (Santa Cruz Biotechnology, Dallas, TX), anti-c-Src, or anti-DAP-12 antibody (Cell Signaling Technology, Danvers, MA) for 30 min, followed by washing and incubation with an FITC-conjugated secondary antibody for 30 min. Confocal microscopic scanning was performed with a Zeiss LSM-510 META laser scanning confocal microscope (Carl Zeiss, Jena, Germany).

### Micro-computed tomography (Micro-CT)

Legs from rats were removed, followed by fixing using paraformaldehyde and decalcification using 20% ethylenediaminetetraacetic acid, which was frequently changed for two weeks, and embedded in paraffin. After serial fixation in 4% paraformaldehyde and 70% ethanol, the bones were scanned by high-resolution micro-CT. Imaging was performed using SkyScan 1076 with a voxel size of 9 μm. Structural parameters were calculated using the SkyScan CT-analyzer to analyze micro-CT datasets for morphometry and densitometry.

### Deoxypyridinoline (DPD) cross-linking assay

Serum DPD levels were measured by urine DPD cross-linked enzyme-linked immunosorbent assay (ELISA) kits according to the manufacturer’s instructions (MicroVue, San Diego, CA). Briefly, conjugated DPD-alkaline phosphatase was added to rat sera from each group as the antibody, and the enzyme activity was detected with a p-nitrophenyl phosphate substrate, the reaction products of which could be detected at absorbance at 405 nm. Elevated levels of serum DPD indicate increased bone resorption in individuals.

### Statistical analysis

Non-parametric Mann–Whitney U test was used for comparison of different parameters between two groups. A *p*-value < 0.05 was considered statistically significant. All analyses were conducted using SPSS software, vers. 16.0 (SPSS, Chicago, IL).

## Results

### TRAIL inhibited RANKL-induced osteoclast differentiation

**Fig. 1:**
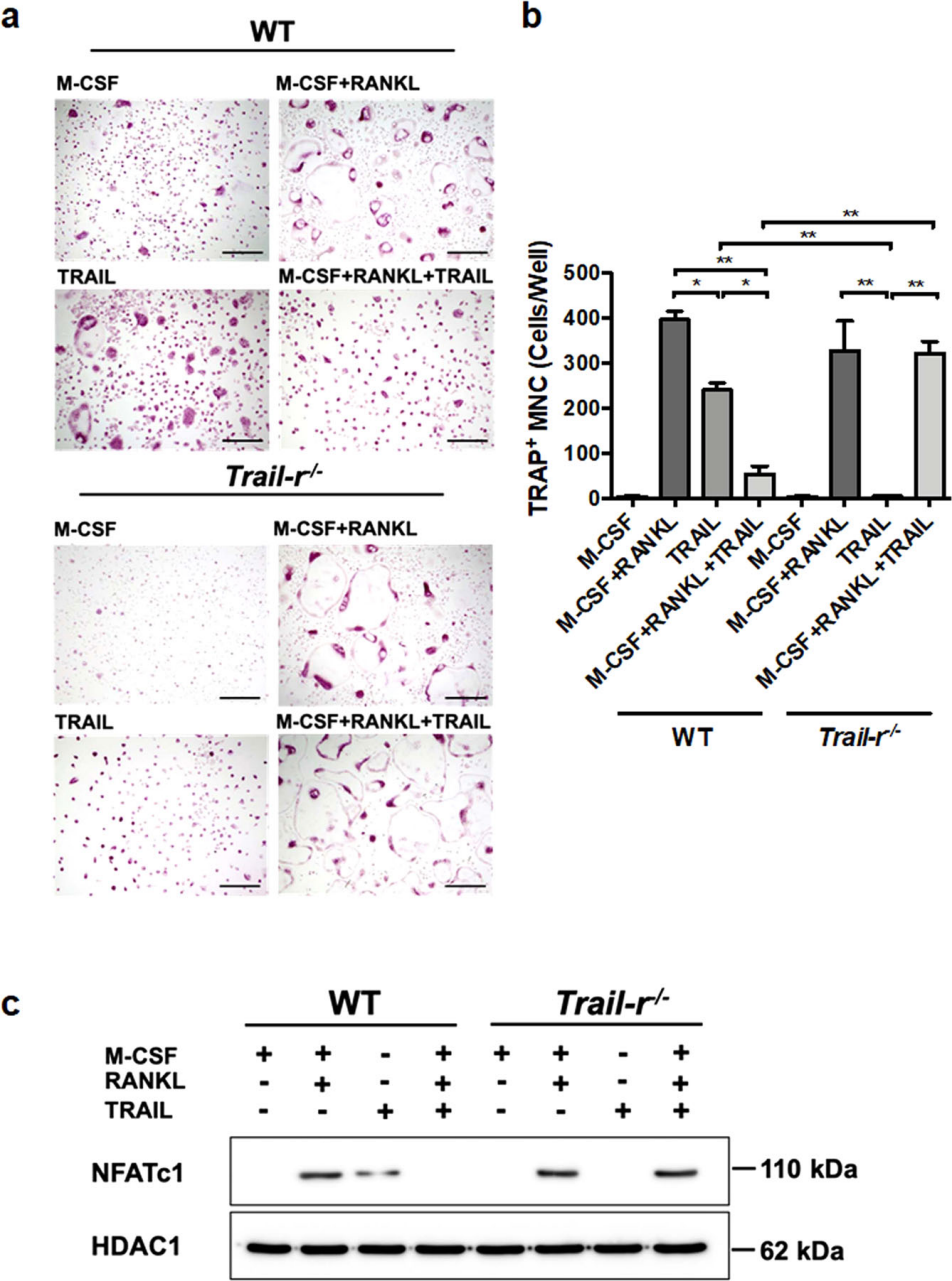
TRAIL inhibits RANKL-induced osteoclast differentiation and activation of NFATc1. **a** Bone marrow-derived macrophages (BMMs) from wild type (WT) and TRAIL-R knockout (*Trail-r*^−/−^) mice were plated in 96-well plates and stimulated with the RANKL (50 ng/ml) + M-CSF (20 ng/ml), TRAIL (500 ng/ml), or RANKL + M-CSF + TRAIL as indicated in the figure. After 10 days, cells were analyzed for osteoclast differentiation. After incubation, cells were subjected to a tartrate-resistant acid phosphatase (TRAP) assay. Cell morphology was examined by light microscopy (Scale bars, 100 µm), and the number of TRAP-positive multinuclear cells was quantified in (**b**). **p < 0.01 by non-parametric Mann–Whitney *U* test. Data are representative of five independent experiments. **c** BMMs obtained from WT and *Trail-r*^−/−^ mice were treated with the TRAIL (500 ng/ml), M-CSF (20 ng/ml), and RANKL (50 ng/ml). After stimulation at 24 h, cells lysates of the nuclear fraction were prepared, and immunoblotted with anti-NFATc1 and anti-histone deacetylase (HDAC) antibodies. The results are representative of three separate experiments

To confirm that TRAIL can regulate RANKL-induced osteoclastogenesis, we first administrated RANKL-pretreated BMMs with TRAIL and analyzed osteoclast differentiation in vitro. Both RANKL (50 ng/ml) plus M-CSF (20 ng/ml) or TRAIL (500 ng/ml) alone could induce BMMs into osteoclasts; but paradoxically when treated with both RANKL plus M-CSF and TRAIL together, the ability to induce osteoclast differentiation by BMMs was markedly inhibited (Fig. 1). TRAIL significantly suppressed the formation of TRAP-positive multinuclear cells (TRAP + MNCs) on RANKL-treated BMMs; and this effect was completely reversed in BMMs from TRAIL-R knockout (*Trail-r*^−/−^) mice (Fig. 1). To further confirm whether RANKL or TRAIL induces osteoclastogenesis signaling through nuclear translocation of NFATc1, the critical transcription factor of osteoclasts, we isolated nuclei to detect the translocation of NFATc1 when cells were treated with RANKL plus M-CSF in the presence or absence of TRAIL. RANKL plus M-CSF or TRAIL alone induced the translocation of NFATc1 into the nuclear fraction of cell lysates. In contrast, TRAIL completely abolished RANKL-induced NFATc1 translocation in osteoclast precursor cells, and this suppressive effect was completely reversed in BMMs from TRAIL-R-knockout mice. To further exclude the possibility that suppression of RANKL-induced osteoclastogenesis by TRAIL is due to triggering apoptosis in osteoclast precursors, we analysed the time course of RANKL plus MCSF stimulated BMMS in the presence or absence of TRAIL in osteoclast differentiation (supplementary figures Fig. S1 and S2), and cell apoptosis during the culture period (Fig. S3 and S4). The results demonstrated TRAIL inhibited RANKL-induced osteoclast differentiation and NFATc1 translocation without triggering cell apoptosis. Moreover, this effect is dependent on interaction with TRAIL-R. All these results indicated that TRAIL completely inhibited RANKL-induced osteoclast differentiation and NFATc1 translocation via an apoptosis-independent pathway.

### TRAIL dissembled RANKL-induced lipid raft-associated signaling complexes

**Fig. 2:**
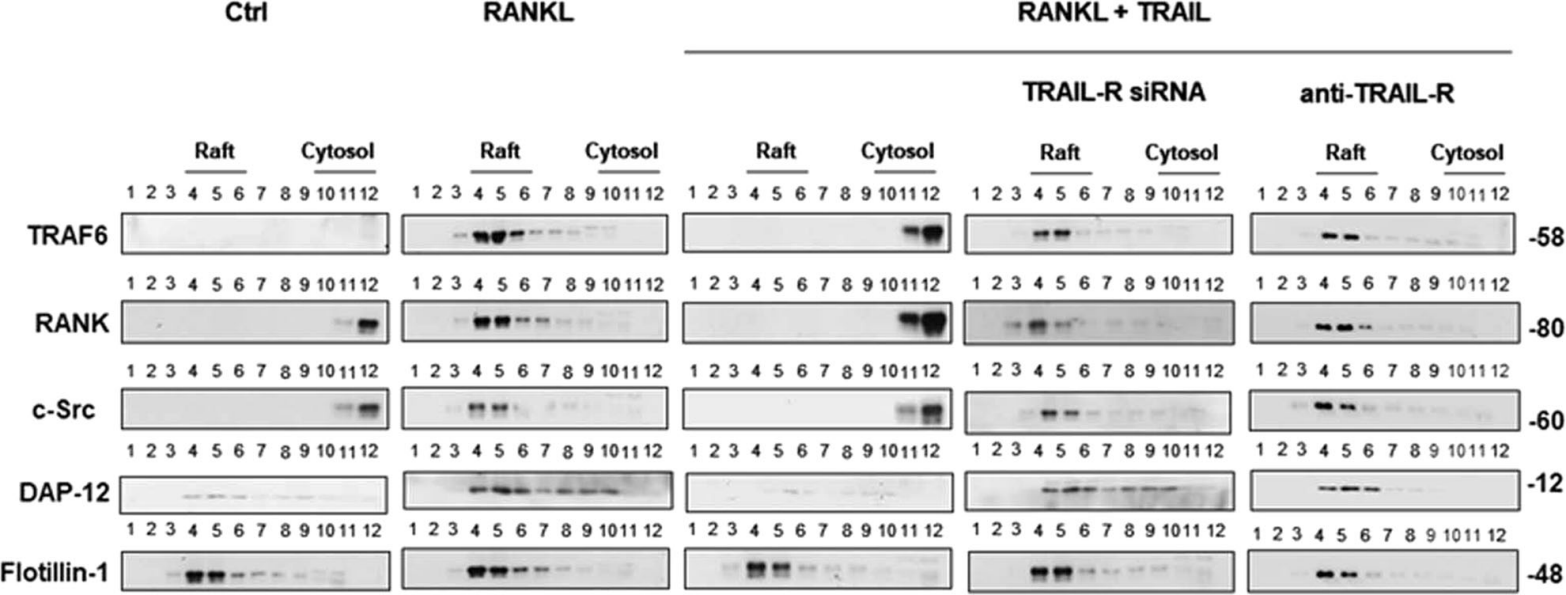
TRAIL dissembled the RANKL-induced lipid raft-associated signaling complexes. RAW 264.7 cells incubated in minimal essential medium were stimulated by the RANKL with or without the TRAIL for 4 h in the presence and absence of TRAIL-R siRNA or an anti-TRAIL-R blocking antibody (Ab). Cells were lysed with TNE buffer containing 0.5% Brij-58 and subjected to sucrose density gradient centrifugation to isolate lipid raft fractions. Proteins from equal volumes of representative collected fractions were separated by SDS-PAGE and immunoblotted with anti-TRAF6, anti-RANK, anti-c-Src, anti-DAP-12, and anti-flotillin-1 Abs. The lipid raft fraction and cytosolic fraction are presented as fractions 4 and 12, respectively

Our results indicated that TRAIL inhibited RANKL-induced osteoclastogenesis and nuclear translocation of NFATc1. RANKL/RANK interaction transduces signaling, followed by a sequential cytoplasmic signaling cascade, and results in nuclear translocation NFATc1, the master transcription factor for osteoclast differentiation^26,27^. Lipid rafts are dynamic assemblies of proteins and lipids that harbor many receptors and regulatory molecules and so act as a platform for signal transduction^23,24^ in immune signaling. It was demonstrated that RANK-mediated signaling and osteoclast function are dependent on the expression and integrity of lipid rafts^25^ (Fig. S5). In order to explore whether TRAIL targets lipid rafts to regulate RANKL-induced osteoclastogenesis signaling, we analyzed the lipid raft composition and distribution of components in osteoclast precursors after stimulation by RANKL in the presence and absence of TRAIL. Lipid rafts are composed of flotillin-1, which serves as a marker for raft fractions. As shown in Fig. 2, when mouse macrophage cell line RAW 264.7 cells were activated by RANKL, it was found that RANK, TRAF6, c-Src, and DAP-12 were recruited into lipid rafts. In contrast, recruitment of these signaling molecules into lipid rafts was abrogated in RAW 264.7 cells activated with RANKL in the presence of TRAIL, suggesting disruption of lipid raft assembly by simultaneous RANKL and TRAIL stimulation.

To elucidate whether TRAIL inhibits RANKL-induced osteoclastogenesis signaling via interaction with TRAIL-R, RAW 264.7 cells were transfected with mouse TRAIL-R siRNA (6 nM) or treated with a TRAIL-R-blocking antibody (2 μg) during induction of osteoclast differentiation. As results demonstrate in Fig. 2, both TRAIL-R siRNA and TRAIL-R blockage completely inhibited lipid raft dissociation effects of TRAIL, indicating that TRAIL exerts its effect through interactions with TRAIL-R.

### TRAIL inhibits the RANKL-induced lipid raft assembly, and is also able to dissociate the raft assembly after its formation

**Fig. 3:**
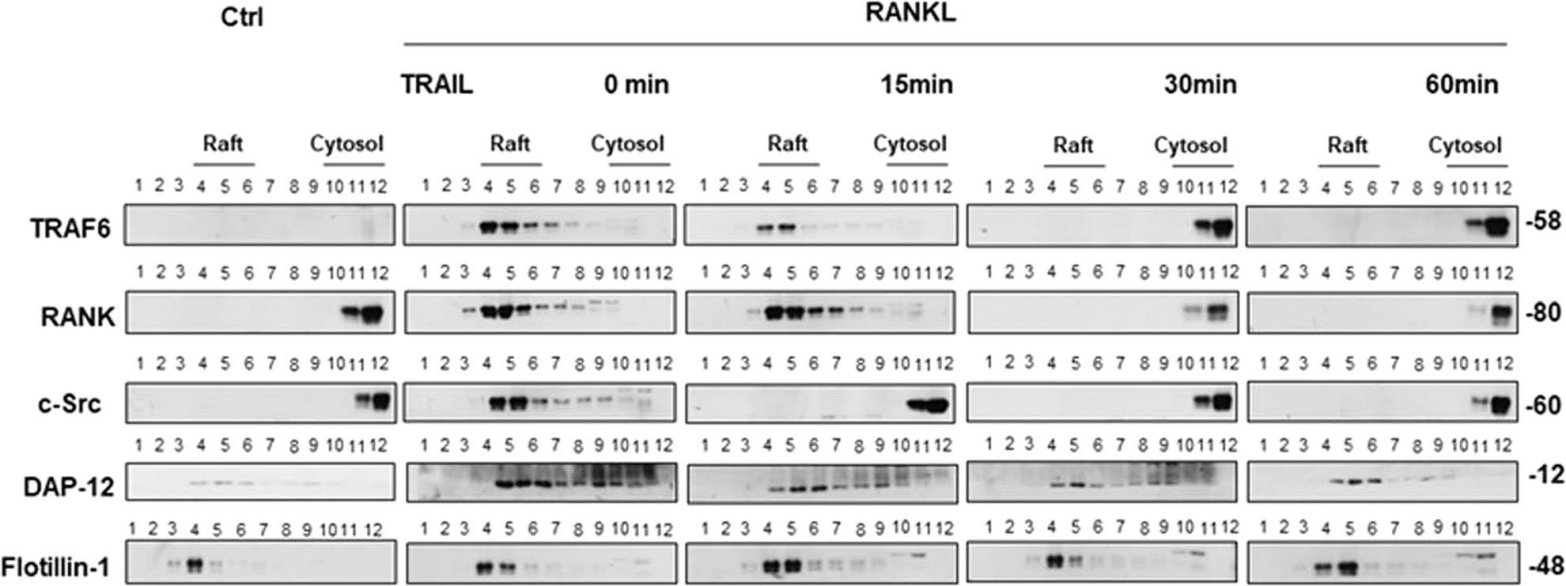
Time course analysis of dissociation of the RANKL-induced rafts associated signaling complexes after its formation by TRAIL. RAW 264.7 cells were incubated in α-MEM medium and stimulated with RANKL and M-CSF for 4 days to induce the osteoclast differentiation. Then, the RAW 264.7-derived osteoclasts were stimulated with TRAIL and collected cell lysates at different time points (0 min, 15 mins, 30 mins and 60 mins). Cell lysates of RAW 264.7 collected at indicated time points were subjected to SDS-PAGE electrophoresis and immunoblotted with anti-TRAF6, anti-RANK, anti–c-Src, anti-DAP-12, and anti–Flotillin-1 Abs. Lysates of cells without stimulation were used for comparison

To understand whether TRAIL is able to prevent RANKL-induced lipid raft assembly or dissociate the assembly after its formation, we stimulated cells with RANKL plus M-CSF for four days to establish the signaling and then treated them with TRAIL after RANKL stimulation, and studied the composition of lipid raft components after TRAIL treatment at each time point. As per results shown in Fig. 3, RANKL stimulation resulted in recruitment of TRAF6, RANK, and c-Src into lipid rafts. At 15 min after TRAIL treatment, TRAF6 and RANK, but not c-Src, were still present in the lipid rafts, indicating partial assembly of the signaling complex. At 30 and 60 min after TRAIL treatment, TRAF6, RANK, and c-Src were absent from raft fractions of cell lysates, indicating that TRAIL-induced dissociation of TRAF6 and RANK from RANKL-induced lipid raft assembly. The raft marker flotillin-1 was still detected in the lipid raft fraction at 15, 30, and 60 min. These results demonstrated that TRAIL is able to disassemble the RANKL-induced signaling complexes from lipid rafts even after it had been formed. Taken together, the results of the lipid raft analysis indicate that TRAIL disrupted the RANKL-induced lipid raft signaling complex either by preventing its formation or dissociating it from lipid rafts after its formation.

### TRAIL inhibited RANKL-induced assembly of lipid raft-associated signaling complexes and TRAF6 recruitment in the confocal image analysis

**Fig. 4:**
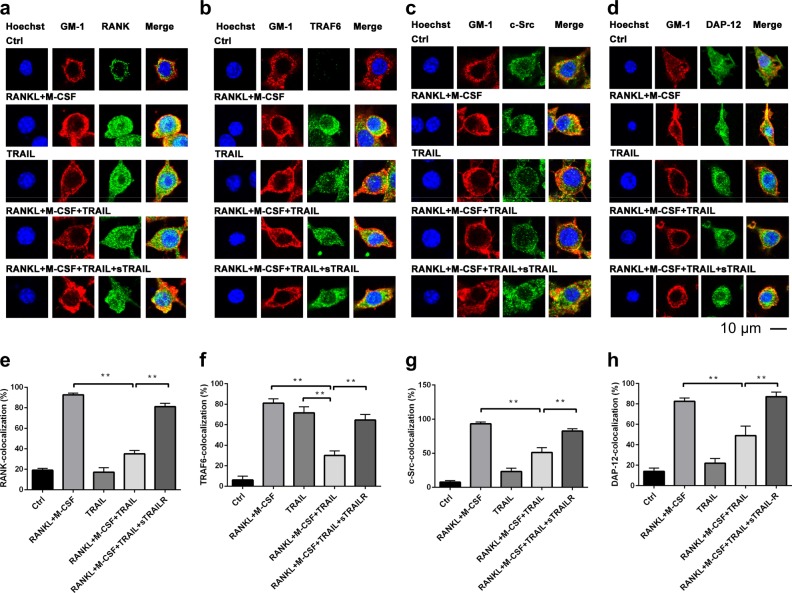
TRAIL inhibition of RANKL-induced co-localization of TRAF6, RANK, c-Src, and DAP-12 in lipid rafts in confocal microscopy. RAW 264.7 cells were cultured on coverslips and stimulated as indicated. After fixation, cells were processed for immunofluorescence staining of cholera toxin B subunit for GM-1 (red), and Hoechst 33258 (blue). Signaling molecules in lipid rafts were examined by confocal microscopy. **a**–**d** The TRAF6 Ab, RANK Ab, Src Ab, and DAP-12 Ab were stained green, and when colocalized with lipid raft GM-1 (red), exhibited yellow staining (merged). (Scale bar represents 10 μm). **e**–**h** The percentages of co-localization of RANK, TRAF6, c-Src, and DAP-12 with lipid raft GM-1 were determined and are shown in the bar chart (measured in at least 80 cells. **p < 0.01 by Mann–Whitney *U* test)

To further confirm the spatial reorganization of lipid raft-associated components on the cell surface of osteoclast precursors after RANKL stimulation, co-localization of these signaling molecules with the raft marker, GM-1, was further analyzed by confocal microscopic imaging. Localization of RANK, TRAF6, c-Src, and DAP-12 in lipid rafts was analyzed in RAW246.7 cells after RANKL stimulation in the presence and absence of TRAIL. Results in Fig. 4 demonstrate that RANK, TRAF6, c-Src, and DAP-12 were not located in lipid rafts when cells were unstimulated. After RANKL stimulation, RANK, TRAF6, c-Src, and DAP-12 were recruited into lipid rafts and colocalized with GM-1 according to the confocal imaging study. In contrast, concomitant treatment with RANKL and TRAIL resulted in decreased co-localization of RANK, TRAF6, c-Src, and DAP-12 within lipid rafts, indicating that TRAIL inhibited RANKL-induced recruitment of RANK, TRAF6, c-Src, and DAP-12 into lipid rafts. To further confirm that this effect was via TRAIL-R, a soluble (s)TRAIL-R was used to block the interaction between TRAIL and TRAIL-R. Results demonstrated that treatment with sTRAIL resulted in abolishment of TRAIL-induced dissociation of RANKL-mediated lipid raft assembly. All these results indicate that TRAIL suppresses the RANKL-induced assembly of lipid raft-associated signaling, and further inhibits RANKL-induced osteoclastogenesis.

### TRAIL suppressed joint damage and protected against bone resorption in rats with CIA

**Fig. 5:**
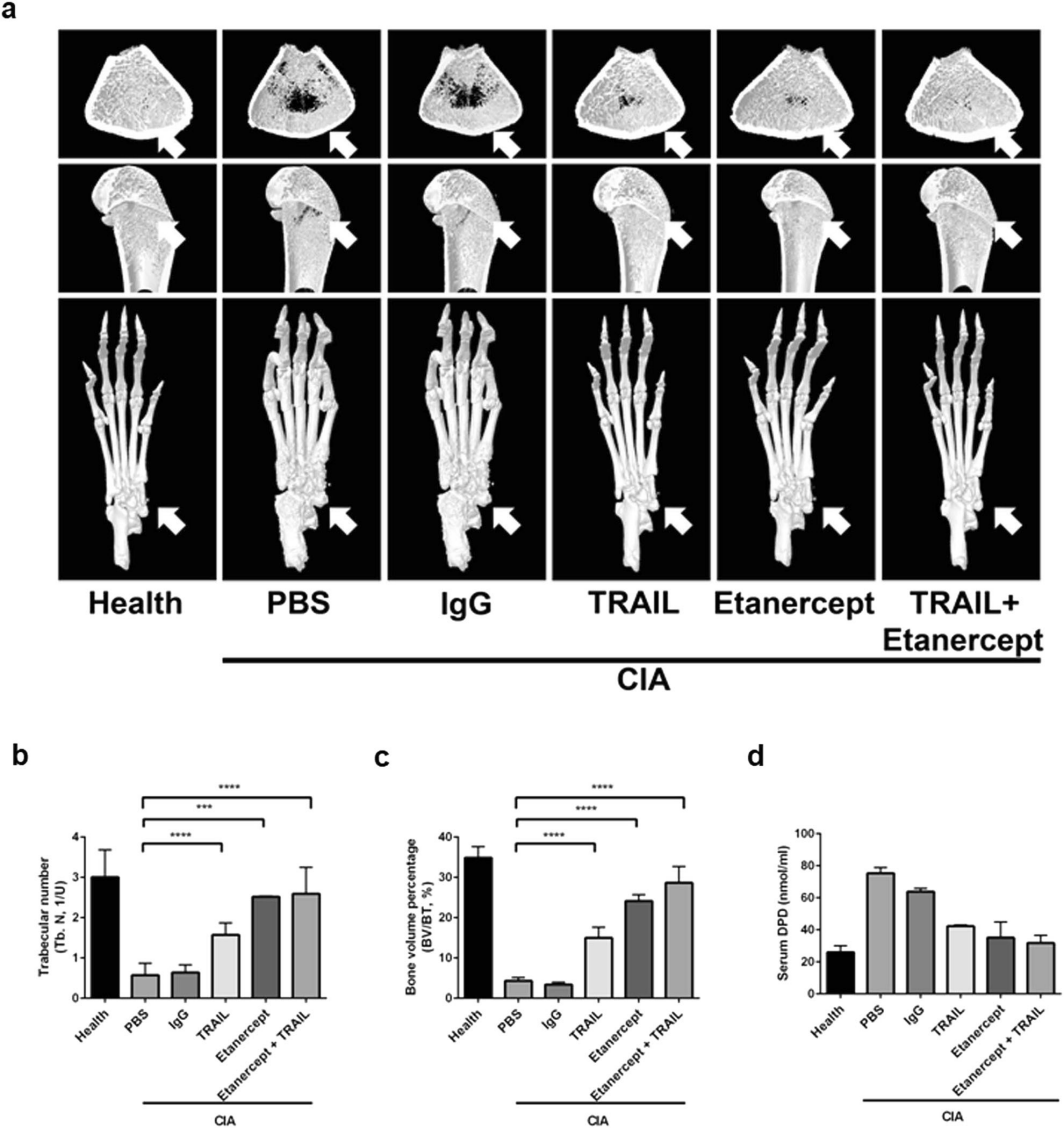
TRAIL suppressed bone erosion and protected against bone loss in rats with collagen-induced arthritis (CIA). The TRAIL or etanercept was injected into the right ankle on day 11 after CIA induction. Femurs and hind paws from rats were harvested on day 29 and prepared for a micro-CT analysis. Blood samples were collected on the sacrifice day for quantification. The trabecular number (Tb.N) and bone volume per tissue volume (BV/TV) were measured by micro-CT in 11 rats with CIA. **a** Representative micro-CT of femurs of rats treated with isotype IgG, TRAIL (1.5 mg/kg/day), etanercept (6 mg/kg three times per week) or etanercept + TRAIL (top, axial view; middle. longitudinal view; bottom, 3D view of hind paws). Arrows indicate the portions of bone erosion compared to isotype IgG. **b** Data of the Tb.N are shown in the bar chart. (*n* = 11; ***p < 0.001, ****p < 0.0001 by Mann–Whitney *U* test). **c** Data of the BV/TV are shown in the bar chart. (*n* = 11; ****p < 0.0001 by Mann–Whitney *U* test). **d** Serum level of DPD (nmol/ml) in CIA rats. DPD in the sample competed with conjugated DPD-alkaline phosphatase for the antibody, and the reaction was detected with a p-nitrophenyl phosphate substrate. The absorbance was measured at 405 nm (*n* = 11)

We further investigated the role of the TRAIL in arthritis-induced bone erosion. Etanercept, a soluble TNF receptor used clinically for treating rheumatoid arthritis, was used as a positive control. A micro-CT analysis revealed that rats with CIA exhibited profound osteolytic lesions with a markedly decreased bone volume density in inflamed paws and femurs on day 29 post-collagen injection (Fig. 5). Significant decreases in the bone volume density and trabecular thickness were observed in femurs of rats with CIA. On the contrary, treatment of rats with the TRAIL effectively suppressed inflammation-induced joint damage, and this effect was comparable to that seen with etanercept treatment. In addition, the level of DPD cross-linked in inflamed paws of rats treated with TRAIL was significantly reduced, revealing marked restoration of the process of inflammation-induced bone resorption. The combination of the TRAIL and etanercept had additional therapeutic effects of protecting against inflammation-induced joint damage. Overall, these results indicated that TRAIL not only inhibited inflammatory arthritis, but also effectively ameliorated joint damage and protected against subsequent bone loss.

### TRAIL inhibited inflammation-induced osteoclast differentiation in CIA

**Fig. 6:**
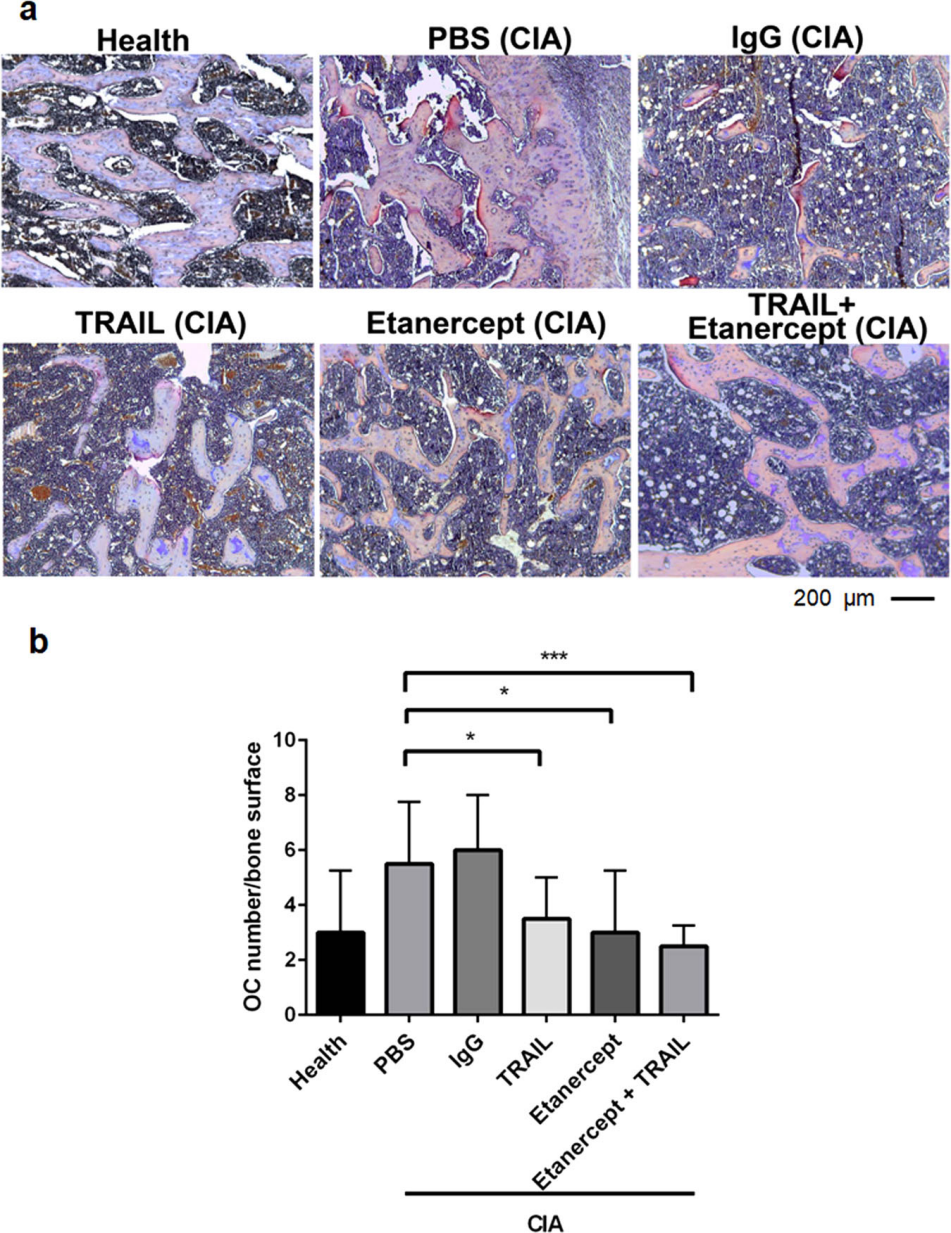
TRAIL inhibited osteoclast formation in rats with CIA. The TRAIL or etanercept was injected into the right ankle on day 11 after CIA induction. Femurs and hind paws from rats were harvested on day 29 and prepared for a histopathological study. **a** Histopathology of the femur subjected to TRAP staining. (Scale bar represents 200 μm; NA, 0.6). **b** The number of osteoclasts (OC) on the trabecular bone surface shown in the bar chart (*n* = 6; *p < 0.05, ***p < 0.001 by Mann–Whitney *U* test)

To further address the in vivo effects of TRAIL on osteoclast differentiation in inflammatory arthritis, we next investigated impacts of TRAIL on osteoclast activation during CIA. As illustrated in Fig. 6, the histopathology of the femur subjected to TRAP staining showed substantial increases in TRAP + osteoclast numbers found in rats with CIA on day 29 post-collagen injection. In contrast, the osteoclast number was significantly reduced in rats with CIA treated with etanercept, TRAIL, or TRAIL plus etanercept on the trabecular bone surface when compared to the controls. Taken together, TRAIL inhibited arthritis-induced joint damage and bone absorption by suppressing osteoclast activation, suggesting a therapeutic role of TRAIL in inflammatory arthritis and regulation of osteoclastogenesis.

## Discussion

In this study, we demonstrate that the TRAIL inhibits RANK signaling and suppresses osteoclast activation via inhibiting lipid raft assembly and TRAF6 recruitment. This suggests a novel apoptosis-independent role of the TRAIL in regulating RANK signaling and suppressing osteoclast activation via inhibiting lipid raft assembly and TRAF6 recruitment. Although the TRAIL induces apoptosis in many tumor cell lines, almost all primary cells are resistant to TRAIL-induced cell death, and the actual biological role of the TRAIL remains to be elucidated. In recent years, accumulating evidence has demonstrated that the TRAIL can modulate immune responses in autoimmune inflammatory diseases via an apoptosis-independent pathway^28,29^. TRAIL administration induced anti-inflammation in several autoimmune animal models^28–32^. All of those studies and our results emphasize the regulatory role of the TRAIL in modulating autoimmune immune responses, and raise potential therapeutic implications of the TRAIL in autoimmune diseases. In the present study, we demonstrated that the TRAIL effectively restored arthritis-induced joint erosion and inhibited bone resorption by suppressing osteoclastogenesis in CIA. These results indicate that the TRAIL is not only potent in inhibiting joint inflammation, but also efficacious in protecting against joint erosion and bone loss in inflammatory arthritis. It evidences a novel role for the TRAIL in regulating osteoclast activation and bone erosion in vivo. Therefore in addition to regulation of immune responses, the TRAIL also regulates osteoclast activation during immune responses, suggesting that the TRAIL may play an important role in osteoimmunology.

It is still not clear how the TRAIL regulates RANK signaling in osteoclast differentiation. In this study, we demonstrated that the TRAIL regulates RANK-induced osteoclastogenesis of the assembly of lipid raft-associated signaling complexes. Previous studies demonstrated that the TRAIL itself induced osteoclast differentiation via activation of a TRAF6-dependent signaling pathway^17,18^; and the TRAIL is also able to suppress osteoclastic differentiation induced by the RANKL plus M-CSF^20^, suggesting that the TRAIL may play a role in regulating osteoclast differentiation. In this study, we further demonstrated that RANKL stimulation induced recruitment of TRAF6, c-Src, and DAP-12 into lipid rafts. However, the RANKL-induced assembly of TRAF6, c-Src, and DAP-12 into lipid rafts was abolished in the presence of the TRAIL. The results also demonstrated that the TRAIL is able to disassemble RANKL-induced signaling complexes in lipid rafts even after they had been formed. This study indicates that the TRAIL disrupts RANKL-induced lipid raft signaling complexes either by preventing their formation or dissociating them from lipid rafts after their formation. The involvement of rafts has been implicated in many important cellular processes, which include cell surface receptor signaling, and the generation and maintenance of cellular polarity. For T cell and B lymphocyte antigen receptors, raft domains function as signaling platforms where selective signaling molecules are recruited^24^. Osteoclast formation from mononuclear phagocyte precursors involves interactions between TNF ligand superfamily members and their receptors^1–4^. However, the signaling pathways and whether lipid raft-associated signaling is essential for RANKL-induced osteoclast differentiation are still not clear. Previous studies demonstrated that raft expression increases during osteoclastogenesis and that TRAF6 is recruited to rafts by RANKL stimulation in osteoclasts^25^. All these findings demonstrate a crucial role for membrane lipid rafts in the function, and potentially differentiation, of osteoclasts. Our study is the first to demonstrate that lipid raft assembly and TRAF6 recruitment are critical for TRAIL-induced suppression of RANK signaling in osteoclast differentiation and bone resorption activity. TRAF6 is the critical RANK-associated signaling molecule regulating osteoclast differentiation and activity. TRAF6-knockout mice displayed a defect in NF-κB signaling and consequently developed osteopetrosis, indicating the essential role of TRAF6 in osteoclast function^7,33^. This study suggested that lipid raft assembly and TRAF6-dependent signaling may be a central pathway in osteoclast differentiation, and the TRAIL modifies RANK signaling by interacting with lipid raft-associated signaling.

In this study, we demonstrated that TRAIL/TRAIL-R interactions transduce signaling, a novel pathway distinct from apoptosis signaling via a caspase cascade, leading to interruption of RANKL-induced lipid raft assembly and TRAF6 recruitment. It is still not clear how the TRAIL-R transduces signals that affect lipid raft-associated signaling. Previous studies by our group demonstrated that the TRAIL directly inhibits T cell activation, and TRAIL/TRAIL-R interactions transduce signals that inhibit phosphorylation of proximal T cell receptor (TCR) signaling-associated molecules, including ZAP70, Lck, LAT, and PLCγ1, with repressed recruitment of these signaling molecules into lipid rafts^29^. These results suggest that the TRAIL-R could transduce signals to modulate the assembly of lipid raft-associated signaling. It provides a novel mechanism of TRAIL-R signaling in regulating osteoclast differentiation, a novel signaling pathway distinct from traditional death receptor signaling.

## Supplementary information

Figure S1

Figure S2

Figure S3

Figure S4

Figure S5
