# Supplementary material for: TRAIL inhibits RANK signaling and suppresses osteoclast activation via inhibiting lipid raft assembly and TRAF6 recruitment
Source: Cell Death Dis. 2019 Jan 28;10(2):77. doi: 10.1038/s41419-019-1353-3 (PMC6349873; doi:10.1038/s41419-019-1353-3)
Supplement: Supplementary file 1 — Figure S1 [file 41419_2019_1353_MOESM1_ESM.pdf]

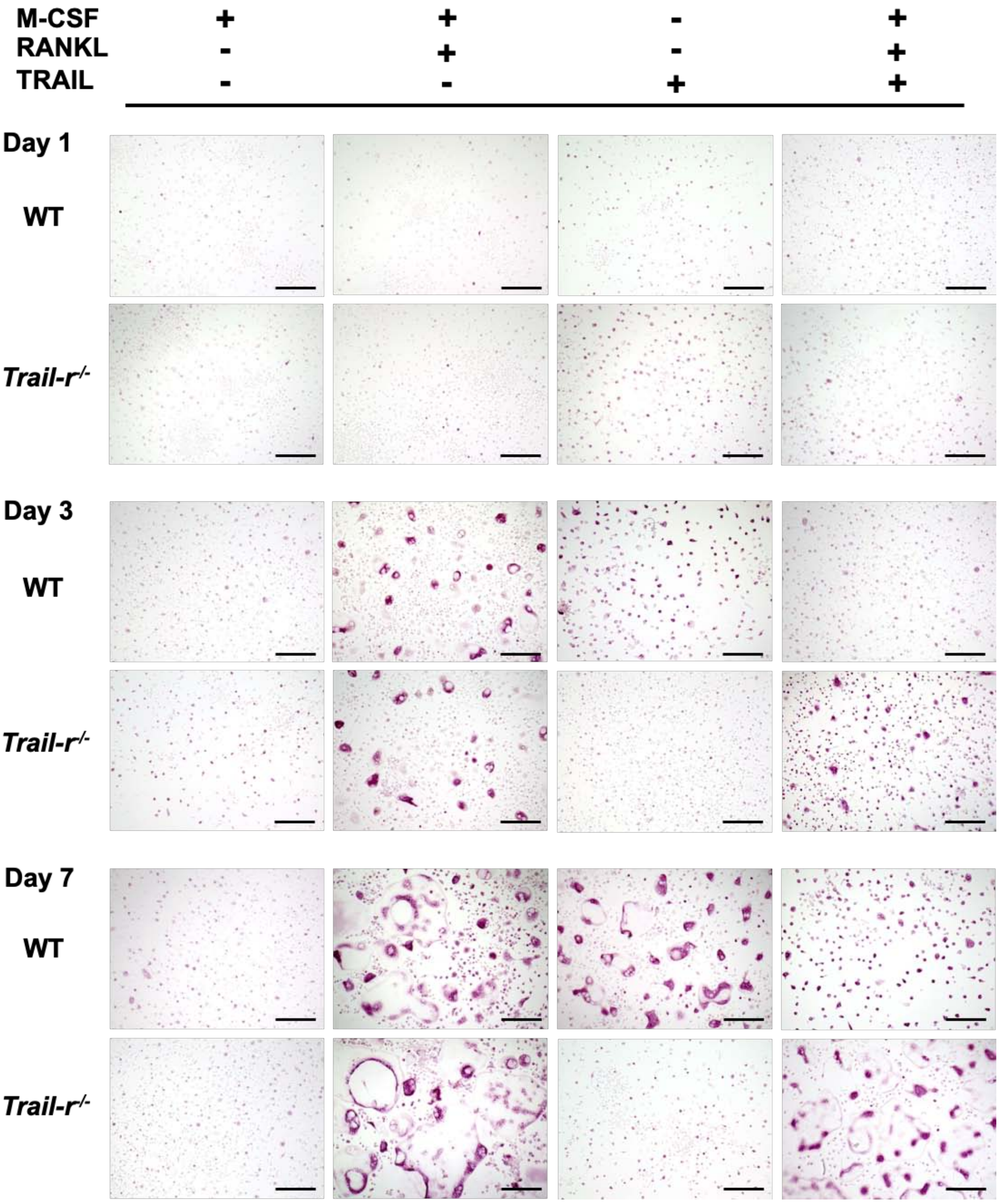
M-CSF + + - +
RANKL - + - +
TRAIL - - + +
Day 1
WT
Trail-r-/-
Day 3
WT
Trail-r-/-
Day 7
WT
Trail-r-/-

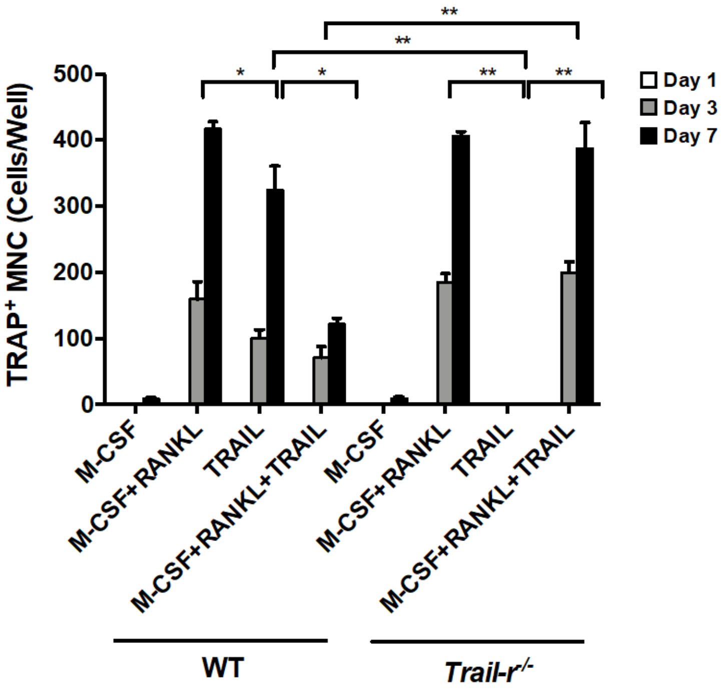
TRAP+ MNC (Cells/Well)
500
400
300
200
100
0
*
*
**
**
**
**
Day 1
Day 3
Day 7
M-CSF
M-CSF+RANKL
TRAIL
M-CSF+RANKL+TRAIL
M-CSF
M-CSF+RANKL
TRAIL
M-CSF+RANKL+TRAIL
WT
Trail-r-/-

Fig S1. TRAIL inhibited RANKL-induced osteoclast differentiation, and this effect is dependent on TRAIL-R

Time-course analysis of osteoclast differentiation induced by RANKL+MCSF, TRAIL, or RANKL+MCSF+TRAIL. Bone marrow-derived macrophages (BMMs) from wild type (WT) and TRAIL-R knockout (*Trail-r*$^{-/-}$) mice were plated in 96-well plates and stimulated with the RANKL (50 ng/ml) + M-CSF (20 ng/ml), TRAIL (500 ng/ml), or RANKL+M-CSF+TRAIL as indicated in the figure. Cells were analyzed for osteoclast differentiation at different time point as indicated. After incubation, cells were subjected to a tartrate-resistant acid phosphatase (TRAP) assay. Cell morphology was examined by light microscopy (Scale bars, 100 μm), and the number of TRAP-positive multinuclear cells was quantified. Representative pictures and number of TRAP-positive multinucleated cells (MNCs) derived from BMMs treated by RANKL + M-CSF, TRAIL or RANKL+M-CSF+TRAIL for 1, 3, and 7 days are shown (*N*=6) .
