# Supplementary material for: TRAIL inhibits RANK signaling and suppresses osteoclast activation via inhibiting lipid raft assembly and TRAF6 recruitment
Source: Cell Death Dis. 2019 Jan 28;10(2):77. doi: 10.1038/s41419-019-1353-3 (PMC6349873; doi:10.1038/s41419-019-1353-3)
Supplement: Supplementary file 2 — Figure S2 [file 41419_2019_1353_MOESM2_ESM.pdf]

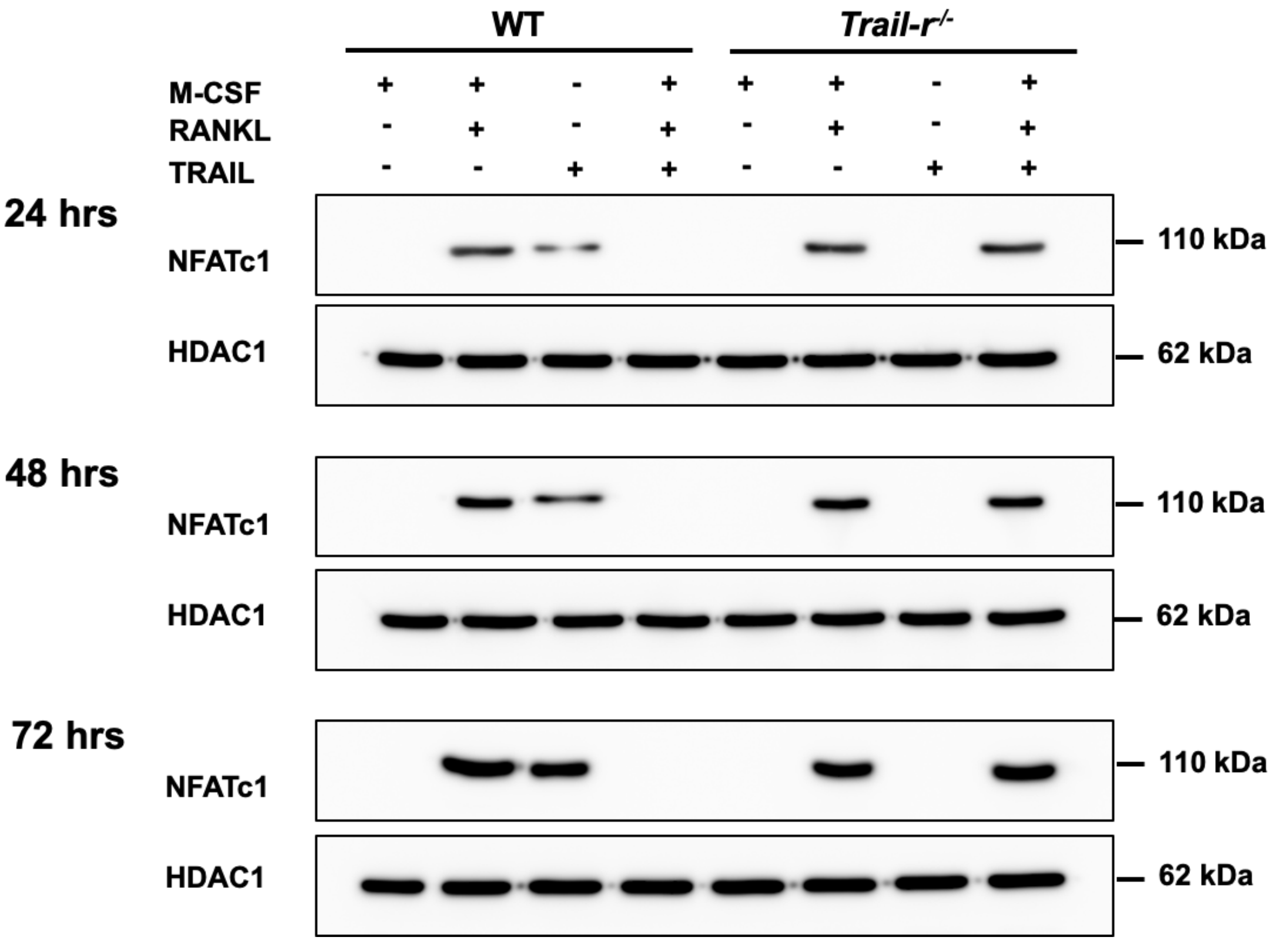

Fig S2 TRAIL completely inhibited RANKL-induced NFATc1 translocation in osteoclast precursor cells, and this suppressive effect was completely reversed in BMMs from *Trail-r* deficiency mice.

BMMs obtained from WT and *Trail-r*$^{-/-}$ mice were treated with RANKL (50 ng/ml) + M-CSF (20 ng/ml), TRAIL (500 ng/ml), or RANKL+M-CSF+TRAIL. After stimulation, cells lysates of the nuclear fraction were prepared, and immunoblotted with anti-NFATc1 and anti-histone deacetylase (HDAC) antibodies. Immunoblot analysis of NFATc1 expression at 24, 48, and 72 hrs after RANKL + M-CSF, TRAIL or RANKL+M-CSF+TRAIL stimulation in the WT and *Trail-r*$^{-/-}$ BMM cell cultures. HDAC1 was used as a loading control.
