# Supplementary material for: TRAIL inhibits RANK signaling and suppresses osteoclast activation via inhibiting lipid raft assembly and TRAF6 recruitment
Source: Cell Death Dis. 2019 Jan 28;10(2):77. doi: 10.1038/s41419-019-1353-3 (PMC6349873; doi:10.1038/s41419-019-1353-3)
Supplement: Supplementary file 3 — Figure S3 [file 41419_2019_1353_MOESM3_ESM.pdf]

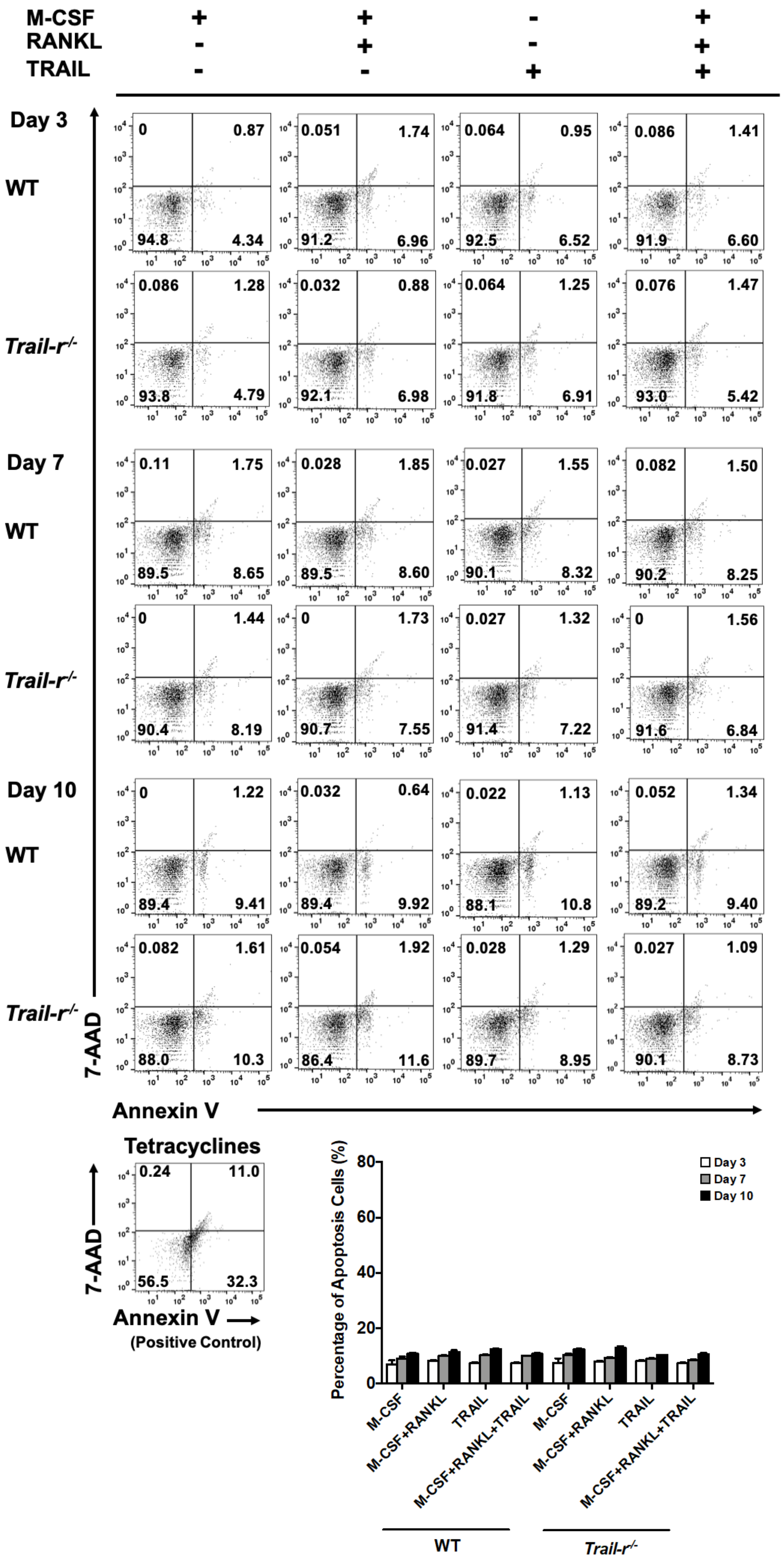

Fig S3. TRAIL did not induce cell apoptosis in RANKL-activated osteoclastogenesis. FACS analysis of osteoclasts after Annexin V/7-AAD double staining for the indicated time. BMMs were cultured with tetracyclines (10 ug/ml) and RANKL (50 ng/ml) + M-CSF (20 ng/ml) for 7 days, for inducing apoptosis. Tetracyclines (10 ug/ml) treatment was used as a positive control. The percentage of apoptosis cells was calculated by FlowJo software of Annexin V fluorescence data (*N* = 6).
