# Supplementary material for: TRAIL inhibits RANK signaling and suppresses osteoclast activation via inhibiting lipid raft assembly and TRAF6 recruitment
Source: Cell Death Dis. 2019 Jan 28;10(2):77. doi: 10.1038/s41419-019-1353-3 (PMC6349873; doi:10.1038/s41419-019-1353-3)
Supplement: Supplementary file 4 — Figure S4 [file 41419_2019_1353_MOESM4_ESM.pdf]

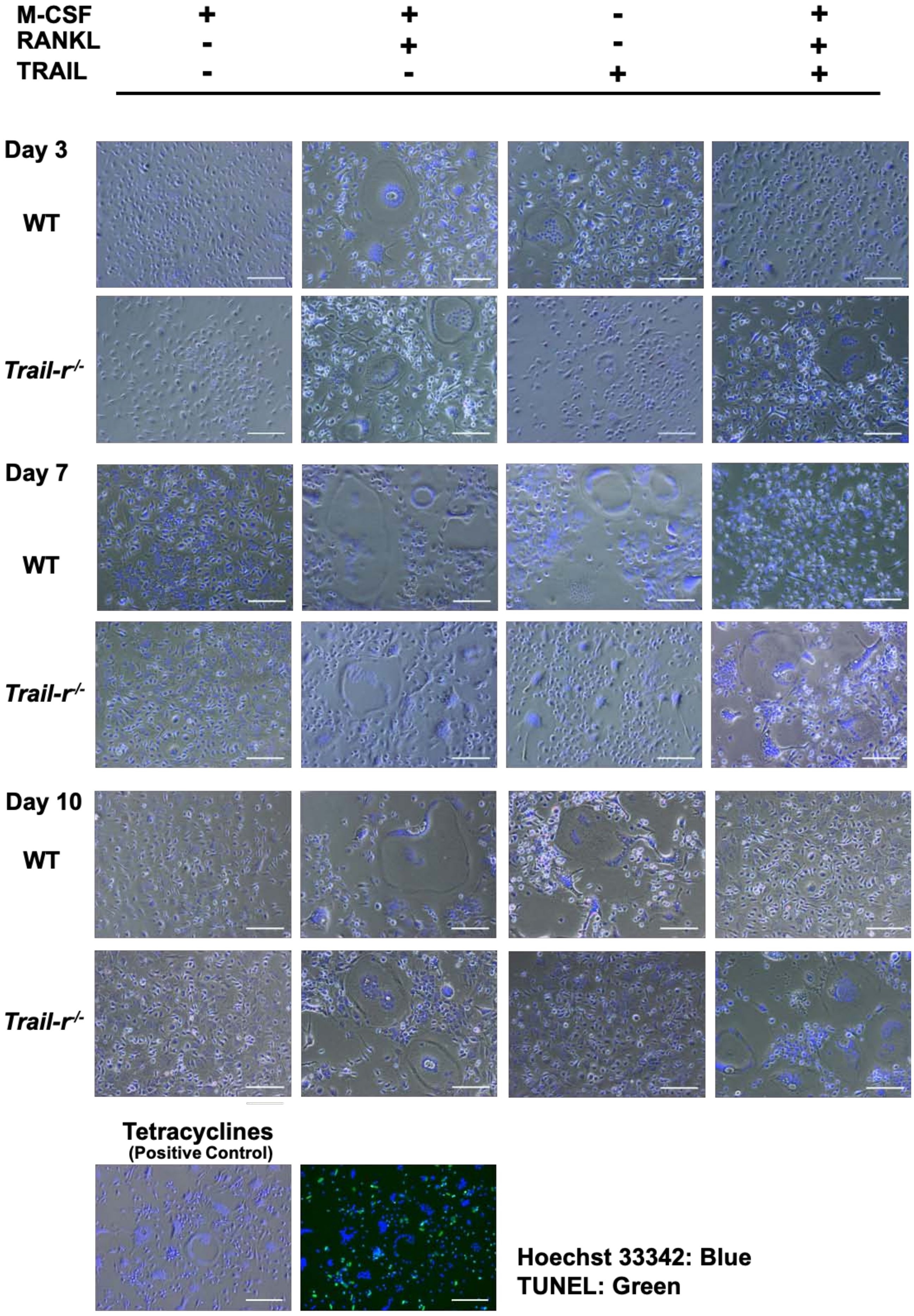

Fig S4. Osteoclast apoptosis is not detected in TRAIL treated RANKL-induced osteoclastogenesis by TUNEL stain.

Differentiation of osteoclasts were cultured with RANKL (50 ng/ml) + M-CSF (20 ng/ml), TRAIL (500 ng/ml) or RANKL+M-CSF+TRAIL for 3, 7, 10 days. Immunostaining followed by fluorescence microscopy revealed the signals from TUNEL (green) and Hoechst 33342 (blue) staining. Tetracyclines (10 ug/ml) treatment was used as a positive control. (Scale bars, 100 μm).
