# Supplementary figures and images for: TRAIL inhibits RANK signaling and suppresses osteoclast activation via inhibiting lipid raft assembly and TRAF6 recruitment
Source: Cell Death Dis. 2019 Jan 28;10(2):77. doi: 10.1038/s41419-019-1353-3 (PMC6349873; doi:10.1038/s41419-019-1353-3)

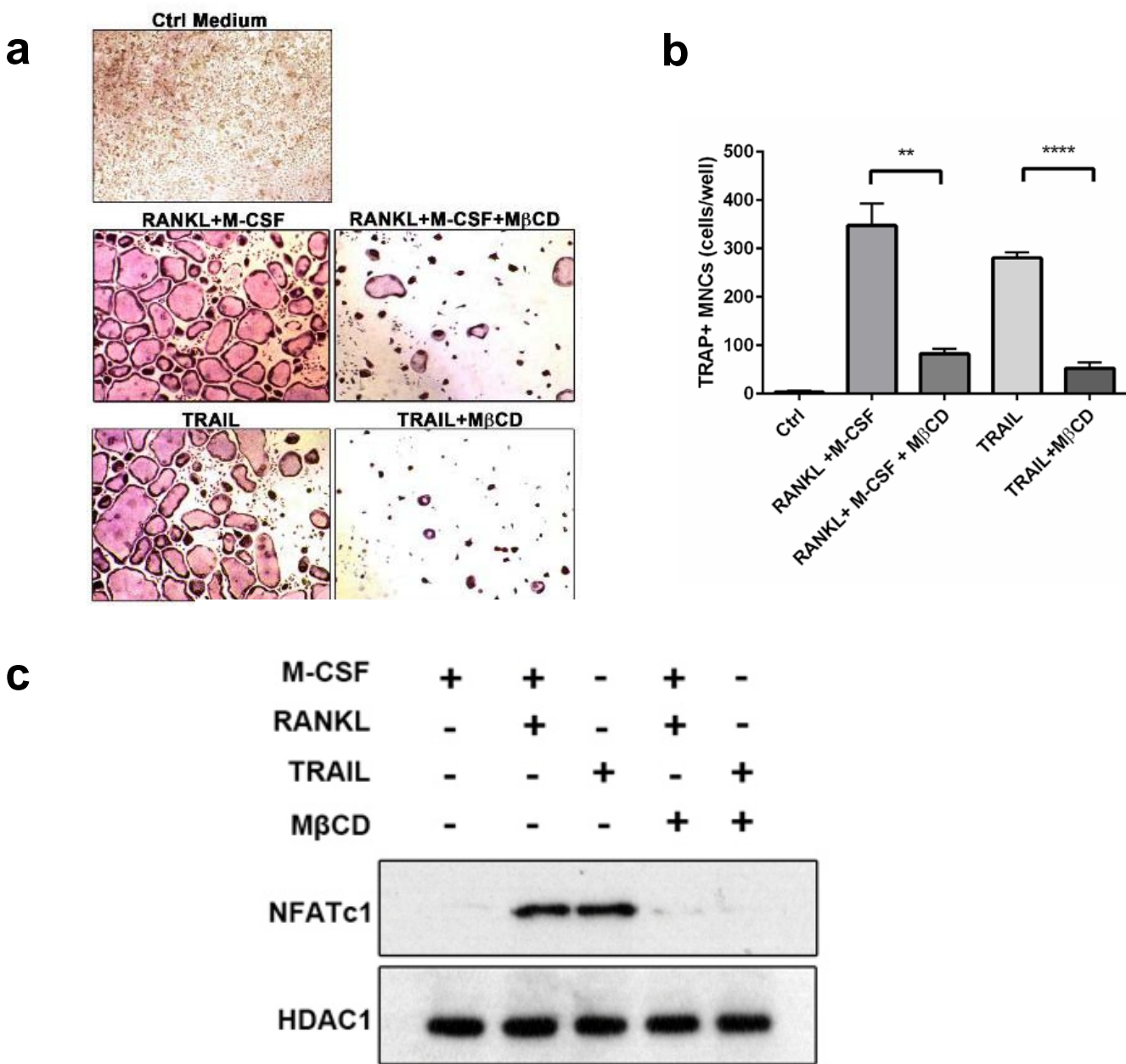

Supplement: Supplementary file 5 — Figure S5 [file 41419_2019_1353_MOESM5_ESM.pdf]
